# Regulation of NADP-Malic Enzyme Activity in Maize (*Zea mays* L.) under Salinity with Reference to Light and Darkness

**DOI:** 10.3390/plants12091836

**Published:** 2023-04-29

**Authors:** Bipul Sarkar, Abir Das, Sayan Pal, Ankita Kundu, Mirza Hasanuzzaman, Masayuki Fujita, Malay Kumar Adak

**Affiliations:** 1 Plant Physiology and Molecular Biology Research Unit, Department of Botany, University of Kalyani, Kalyani 741235, West Bengal, India; bipulsarkar007@gmail.com (B.S.); abir.research@gmail.com (A.D.); psayan693@gmail.com (S.P.); ankitajijibisha@gmail.com (A.K.); 2 Department of Agronomy, Faculty of Agriculture, Sher-e-Bangla Agricultural University, Sher-e-Bangla Nagar, Dhaka 1207, Bangladesh; 3 Laboratory of Plant Stress Responses, Faculty of Agriculture, Kagawa University, Kita-Gun, Kagawa, Miki-cho 761-0795, Japan

**Keywords:** organic acids, allosteric regulation, C4 photosynthesis, proline, photosynthates

## Abstract

We revealed the functional characterization of C4-NADP-malic enzyme (NADP-ME), extracted and partially purified from maize (*Zea mays* L. cv. Kaveri 50). The leaf discs were previously activated under 1000–1200 µE m^−2^ s^−1^, incubated in bicarbonate (2.0 mM) solution, and subjected to salt stress (100 mM NaCl). Initially, salt stress was evident from the accumulations of proline, chlorophyll content, carbohydrate profile, and Hill activity influencing the C4 enzyme. Primarily, in illuminated tissues, the activity of the enzyme recorded a reduced trend through salinity irrespective of light and darkness compared to the control. On illumination, the kinetic parameters such as Vmax of the enzyme increased by 1.36-fold compared to in the dark under salinity whereas Km was decreased by 20% under the same condition. The extent of light induction was proportionate to limiting (0.01 mM) and saturated (4.0 mM) malate concentrations for enzyme activity. Moreover, the catalytic properties of the enzyme were also tested on concomitant responses to activator (citrate and succinate) and inhibitor (oxalate and pyruvate) residues. The sensitivity to light and dark effects was also tested for reducing agents such as dithiothreitol, suggesting the effect of the changes in redox on the regulatory properties of the enzyme. The ratio of enzyme activity under light and darkness in the presence or absence of a reducing agent was concomitantly increased with varying malate concentrations. At the molecular level, protein polymorphism of the enzyme represented minor variations in band intensities, however, not in numbers through salinity subjected to light and darkness. Therefore, salinity-induced changes in the decarboxylation reaction, evident by NADP-ME activity, may be based on the redox property of regulatory sites and sensitivity to light and darkness.

## 1. Introduction

C_4_ photosynthesis is mostly accredited with the bypass of photo respiratory loss of CO_2_, and with the specialization of structural and physiological integrity of tissue diversification (mesophyll cells and bundle sheath cells in leaves). The demarcation of these two types of cells is focused on two distinct functional operations with gaseous CO_2_, primarily with carboxylation in mesophyll cells, and secondarily decarboxylation in bundle sheath cells [1]. For the latter, it is the decarboxylating enzyme that causes the release of CO_2_ from C_4_ acids (malic acid), thereby concentrating the bundle sheath cells with CO_2_. Thus, the released CO_2_ would be the substrate for RuBP-carboxylase in the tissue carrying out the Calvin cycle. Out of the major decarboxylating enzymes, it is the NADP-malic enzyme (NADP-ME) that is predominant both in photosynthetic and non-photosynthetic tissues, and that displays other roles in plant growth and development [2]. NADP-ME is an oxidoreductase [oxaloacetate decarboxylating] that catalyzes the release of CO_2_ with the regeneration of the reducing equivalent as NADPH + H^+^. The NADP-ME is significantly dependent on light flux within photosynthetically active radiation where the photosynthetic form in green tissues contributes a major regulatory role in C_4_ acid partition within the mesophyll cell and bundle sheath cell. In different studies, the importance of malate metabolism has strongly been advocated for, not only as a predominant source of carbon assimilation through different residues but also in other physiological functions, particularly in stress avoidance [3]. In those studies, the close relationship with the NADP-ME activity and the allocation of photosynthates was established as a compensatory mechanism for tissue dehydration under moisture stress or allied salinity stress [4]. Contrarily, under close stomatal condition in moisture stress, the flux of organic acid export from the mesophyll cell to the bundle is facilitated as a strong compensatory mechanism when CO_2_ uptake is restricted. Moreso, when stomata are closed under salinity, the accumulation of excess NADPH may be a factor for the induction of oxidative stress particularly under high irradiance. The NADP-ME would also be operative under this condition through the release of CO_2_ in optimum conditions that might be reduced by NADPH and ATP. Thus, the clarification of C_4_-specific NADP-ME is justified as a better control valve for photo-oxidation in mesophyll tissue [5].

The NADP-ME establishes other important roles in the control of metabolic flux, particularly for those metabolites originating from primary photosynthates. This is more aimed at the conversions of photosynthates into more complex sugars and other biomolecules. These include not only the sucrose–starch interconversion but also the biosynthesis of chlorophyll and other pigments. NADP-ME, with its sustained activity for development of a saturated CO_2_ pool, also induces the primary photochemical reactions such as Hill activities. In turn, the latter in oxygenic photosynthesis undoubtedly develops a thrust for the photochemical quenching of absorbed light in the reaction center, and this results in a non-depleted pool of reducing equivalents. NADP-ME, irrespective of its localization either in a chloroplast or bundle sheath for C_4_ or the cytosol of CAM and C_3_ members, opens a path for CO_2_ supplementation, which is required in carboxylation by Rubisco [6]. Moreover, NADP-ME in a counter path with a major carboxylase, PEP carboxylase (PEPC), controls the intercellular pH in two sub-cellular fractions, chloroplast and cytosol, by a malate transporter. Besides the direct involvement in malate metabolism, this enzyme also meets the requirements for reducing potential as NADPH + H^+^ for the biosynthesis of stress resistance residues, which requires carbon and energy from photosynthetic pools [7].

A few numbers of metabolites including glyceraldehyde-3-phodphate hydrogenase, sedoheptulose 1,7-bis phosphatase, ribulose 5-phosphatase, fructose 1,6-bis phosphatases, etc., are regulated by the reactions of NADP-ME with light regulatory modalities [8]. There are a few other enzymes which are dependent on light activation of NAD-ME and thereby its product NADPH + H^+^ and those are C_4_ specific pyrophosphatase dikinase and NADP-dependent malate dehydrogenase. To the best of our knowledge, the over expression of NADP-ME, and of its transcript and enzyme activity, is based on light-induced de novo protein synthesis. For C_4_ cereal species, the greening of the leaves and shoot and translocation of photosynthates to root is also induced by light-activated NADP-ME activity. This has been demonstrated with the application of an activator and inhibitor of NAD-ME on a light- or dark-adapted leaf disk. This is also reflected in preliminary stress resistance to dehydration or salinity in C_4_ seedlings. Therefore, sustainability or stress resistance may be dependent partially on the activity of NADP-ME under light and dark influence. Moreso, there has been a good correlation recorded with salt tolerance under NaCl [9], toxic metal contamination with the development of special metabolites (proline, glycine betaine) and antioxidants (ascorbate, glutathione). Therefore, the objective of the paper is precisely to find out a few details about the biochemical regulation of NADP-ME under light and darkness as an inducer and to evaluate the impact of salinity thereon. Additionally, the exercise of elicitors such as activators and inhibitors could be important in order to decipher the actual regulatory steps for the enzymes, which is another important concern. The study corroborates the dependence on malate metabolism and its resultant effects on NADP-ME activity in plant systems under light, darkness, and salinity.

## 2. Materials and Methods

### 2.1. Plant Materials and Seedling Development

Seeds of the maize (*Zea mays* L. cv. Kaveri 50) were thoroughly sterilized with 0.1% sodium hypochlorite (NaOCl) for 15 min following washing under tap water several times. Seeds were spread on wet cheese cloth socked with distilled water and transferred to a germination rack in a seed germinator (BTR Kolkata, LAB-X) with the maintenance of optimum conditions. The 15-day-old seedlings with 3–4 leaves were selected for proper experimentation, and were transferred into nutrient solution with 1/4th strength of MS media [10] for 7 days under normal daylight conditions. The depletion of the nutrients was controlled by changing the solution every alternative 3 days, and plants were kept under a 12 h photoperiod maintaining 35 °C (day)/30 °C (night) and with adequate relative humidity.

### 2.2. Salt Treatment

The plants were divided into two groups: one transferred to 100 mM of NaCl solution and another to 0 mM NaCl (control) solution, dissolving 1/4th MS media for 48 h under natural conditions. The treatment was maintained with a minimum of three biological replicates distributed with plants in a completely randomized block design. The effective concentration of NaCl executed for the experiment was standardized against seedling survival in laboratory conditions.

### 2.3. Induction of Enzyme Activity by Light (L)/Darkness (D) and Bicarbonate Treatments

The fully grown second leaf of the main shoot of plants was cut into leaf discs (0.5 cm^2^) underwater in the morning (11 am–12 pm) for each treatment. One set with leaf discs was transferred into Petri plates containing 20 mL 2.0 mM sodium bicarbonate (NaHCO_3_) (as standardized herein) dissolved in distilled water and irradiated with 1000–1200 μE m^–2^ s^–1^ under a fluorescent lamp for 35 min, as standardized for optimum enzyme activity in the present experiment. Another set containing leaf discs treated the same way was placed under complete darkness for the same period. After incubation, immediately, leaf discs were frozen in liquid nitrogen and stored in −80 °C for the assay of enzymes and related biochemical parameters.

### 2.4. Extraction and Purification of NADP-ME Enzyme

The uniform size of the leaf disc from the illuminated or dark-adapted set was thoroughly crushed in liquid nitrogen into a fine powder. The powder was mixed with extraction buffer containing 100 mM Tris-HCl (pH 7.3), 1.0 mm EDTA, 1.0 mM PMSF, 2% glycerol, 100 µM β-ME, 25 mM MgCl_2_, 20 mM K_2_HPO_4_, and 0.1 mM protease inhibitor cocktail as the modified form [11]. The mixture was centrifuged at 12,000× *g* for 15 min at 4 °C and the supernatant was subjected to 80% ammonium sulphate cut, overnight at 4°C. The pellet was recovered and separated by a cellophane tube (50K MWCO) in the same buffer mixed with 0.05% SDS, 25 µM BSA, 0.5 mM MgCl_2_, and 1 mM DTT. Finally, the concentration of soluble protein was checked by Bradford reagent [12] against BSA as a standard.

### 2.5. Assay Buffer and Reaction for NADP-ME Activity In Vitro

The concentrated protein was mixed with an assay mixture of 1 mL containing 50 mM Tris-HCl (pH 7.8), 1 mM MgCl_2_, 10 mM EDTA, 1 mM sodium salt of NADP, and 100 µg equivalent of protein extract. For the substrate L-malate, two sets of assay mixture were taken: 4.0 mM of malate (saturated) or 0.01 mM of malate (limiting), as recommended by Murmu et al. [13]. In each set, 4 mM of citrate, as well as succinate (activators), 1 mM pyruvate, and oxalate (inhibitors) were used to observe the variations in enzyme activities under allosteric regulation. Finally, the activity of enzyme was measured by recording the changing absorbance at 340 nm read with a UV–vis spectrophotometer (Cecil, CE 7200) for 3 min with 30 sec intervals, using an extinction coefficient of 6220 M^−1^ cm^−1^ and expressed as nM NADPH produced min^−1^ mg^−1^ protein.

### 2.6. Separation and Staining of Polymorphic Isoenzyme Bands of NADP-ME

For the separation of polypeptides for NADP-ME, electrophoresis was performed using 6% (*w*/*v*) polyacrylamide gel without any denaturing agents. Partially purified protein was run as 100 µg lane^−1^ under 110 V in cold conditions, with a running buffer of 65 mM Tris-glycine (pH 7.8). The *In-gel* staining of isoenzymes was performed by immersing them in a solution containing 50 mM Tris-HCl (pH 7.5), 15 mM L-malate, 5 mM MgCl_2_, 0.5 mM NADP, 100 µg mL^−1^ phenazine methosulfate, and 0.5 mg mL^−1^ nitroblue tetrazolium disodium salt, and keeping them in the dark for 2 h [14]. The bands were resolved by repeatedly washing the gel under deionized water, and the images were captured in a gel documentation system (Bio-Rad, Gel Doc XR+, Hercules, USA), and densitometry scanning was performed using Gel Analyzer software (Gel Analyzer 10a).

### 2.7. Estimation of Chlorophyll Content and Determination of Hill Activity

Chlorophyll was extracted from 500 mg of tissue, crushed in 80% methanol, filtered, and the absorbance was read at 652 and 665 nm using a UV–vis spectrophotometer (Cecil, CE 7200). Finally, the chlorophyll (*a* + *b*) content was calculated using the following formula, as suggested by Liang et al. [15]:

Chlorophyll (*a* + *b*) = 22.12 A652 + 2.71 A665

The activity of photosystem II was determined by measuring the 2, 6-dichlorophenolindophenol (DCPIP) reduction, with the initial illumination of intact leaves for 2 h. Tissues were homogenized in 50 mM Tris-HCl (pH 7.8) in cold conditions and subjected to sieving through a 3-layer cheese cloth. The filtrate was collected and added to 10 mM sorbitol in a buffer containing 10 mM NaCl, 5 mM MgCl_2_, 10% sucrose, 5% glycerol (*v*/*v*), and was then centrifuged at 8000× *g*, 4 °C for 12 min. The pellet was saved and re-suspended in the same buffer containing 10 mM sorbitol, and immediately stored on ice. In the assay mixture, a chloroplast suspension equivalent to 10 mg mL^−1^ of chlorophyll content was added to the reaction medium in the same buffer containing 0.5 mM DCPIP, kept under light (1000–1200 µE m^−2^ s^−1^) for 3 min, and the absorbance was read at 580 nm. Control sets were performed where the chloroplast and light were withdrawn under the same condition. The activity was measured using the formula as suggested by Sawhney and Naik [16] and expressed as µg DCPIP reduced mg chl^−1^ h^−1^.

### 2.8. Determination of Reducing Sugar and Total Sugar Content

To determine reducing sugar, dry sample was extracted in 70% hot ethanol and crystalline phenol was added with 2, 4-dinitrosalicylic acid (DNS) reagent in a ratio of 5:1 in 1% NaOH. For dilution, 40% Rochelle salt was added and the absorbance was read at 520 nm by a UV–vis spectrophotometer (Cecil, CE 7200) [17].

For total carbohydrate determination, the dry sample was hydrolyzed in 5.0 mL of 2.5 N HCl for 3 h and made neutral with sodium carbonate powder. The extract was centrifuged at 10,000× *g* for 10 min, and the supernatant was mixed with 0.2% anthrone reagent. The reaction mixture was boiled for 30 min and read at 630 nm. Dextrose solution was used as the standard, and the content of sugar was expressed on a fresh weight basis [18].

### 2.9. Quantification of Proline Content

Proline content in tissue was determined through homogenization in 3% sulfosalicylic acid and soup was collected from centrifugation at 12,000× *g* for 15 min at 4 °C [19]. Then, 1 mL of solution was mixed with acid ninhydrin–glacial acetic acid (1:1) and boiled at 100 °C for 1 h. After cooling, 4 mL of toluene reagent was added into the mixture, vortexed well, and then the upper aqueous layer was aspirated and the absorbance was read at 520 nm. L-proline was used as the standard within the linear range of 10–500 µM mL^−1^ to deduce the concentration of the sample and was expressed as µM g^−1^ fresh weight.

### 2.10. Determination of Na^+^ and K^+^

Sodium and potassium (Na^+^ and K^+^) contents were determined according to Rashed [20] through atomic absorption spectrophotometric (AAS) (Varian AA240) analysis. Dried leaf samples were powdered and digested with tri-acid mixture (H_2_SO_4_:HNO_3_:HClO_4_ = 3:3:1, v/v/v) until the solution became clear. Then, the solution was filtered followed by proper dilution and it was used for AAS.

### 2.11. Determination of Malic Acid and Pyruvic Acid Content through High-Performance Liquid Chromatography (HPLC)

A total of 1.0 g leaf tissue was extracted with 80% methanol and filtered through a nylon membrane filter (0.22 µm), Thermofisher Scientific, Waltham, USA, and subjected to HPLC analysis (Agilent 1260 equipped with a diode array detector (DAD), Agilent Technologies, Santa Clara, USA). A Luna C18 column (4.6 × 250 mm) (Phenomenex, Torrance, USA) (set at 25 °C) coupled with reversed-phase (60% acetonitrile with 0.1% acetic acid as solvent) was used with a retention time of 7–8 (malic acid) min and 3–3.5 (pyruvic acid) min, with a constant flow rate of 1.0 mL min^−1^, sample injection volume of 20 µL, and was detected at 210 nm by the DAD. The malic acid and pyruvic acid contents were analyzed and calculated from the chromatogram, with standards of malic acid and pyruvic acid (sodium pyruvate) from the recorded peak area through OpenLab Chem Station software (Agilent Technologies, Santa Clara, USA) [21].

### 2.12. Statistical Analysis

The data were represented herein as mean ± SE (n = 3) from three independent replicates. Significant differences were calculated among and between treatments through the Tukey honestly significant difference (HSD) test using XLSTAT v.2021.

## 3. Results

### 3.1. Inductions of NADP-ME under Light and Darkness

**Figure 1:**
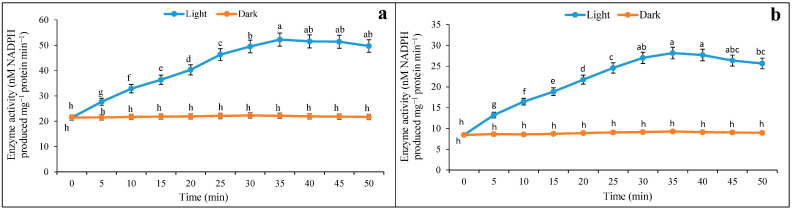
The optimum activity of NADP-ME leaf discs under saturated (**a**) and limiting (**b**) malate concentrations when illuminated with light and darkness under 2 mM HCO_3_^−^ solution through 50 min of incubation. The assay was made from 3 different sets of experiments and data were represented by mean value ± SE. Different small letters on bar indicate significant at *p* ≤ 0.5.

**Figure 2:**
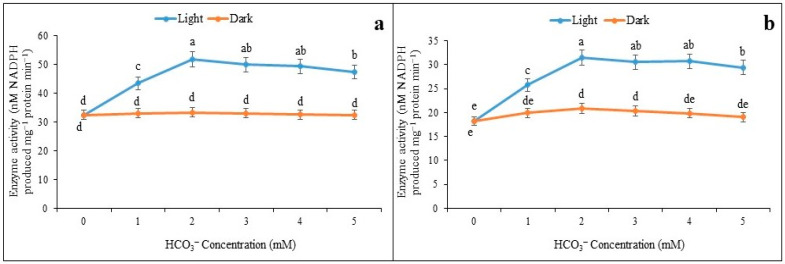
The activity response curve of NADP-ME leaf discs under saturated (**a**) and limiting (**b**) malate concentrations through varying concentration of HCO_3_^−^. The assay was made from 3 different sets of experiments and data were represented by mean value ± SE. Different small letters on bar indicate significant at *p* ≤ 0.5.

The 14-day-old maize seedlings were taken for experimentation and leaf discs (0.5 cm^2^) were illuminated under white light 1000–1200 µE m^−2^ s^−1^. The optimum induction of enzyme activity was obtained at 35 min through an exposure duration of 50 min, irrespective of saturated (4.0 mM) and limiting (0.01 mM) of malate concentration (Figure 1a,b). It is interesting to note that limiting and saturated malate concentration had almost equally optimized the enzyme activity at 35 min. On the other hand, it is observed that NADP-ME activity increased proportionately from leaf discs incubated under a light intensity of 1000–1200 µE m^−2^ s^−1^. The activity of enzymes was recorded as being increased; however, this happened with 2 mM of HCO_3_^−^ solution both under limiting and saturated malate concentrations (Figure 2a,b). The inductions of NADP-ME activity under light by HCO_3_^−^ had almost a similar trend under saturated and limited malate concentration, and probably indicated the regulatory mechanism of the enzyme with the photosynthetic irradiation.

### 3.2. Activators and Inhibitors Affect the Regulation of Enzyme Sensitivity under Light and Darkness

**Figure 3:**
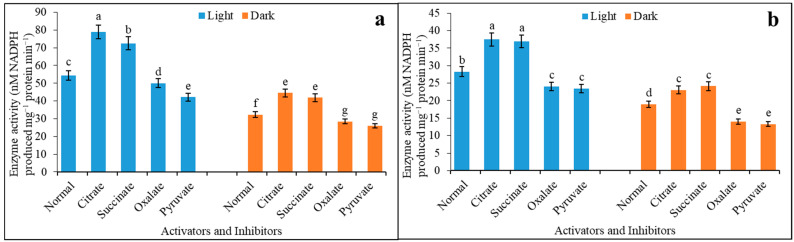
NADP-ME activity with activator and inhibitor under light and darkness in 4.0 mM (**a**) and 0.01 mM (**b**) malate concentration. Leaf discs were cut in equal size and incubated with 2 mM HCO_3_^−^ and thereafter, treated with light and darkness. Leaf discs from each treatment were incubated in activator (citrate and succinate) and inhibitor (oxalate and pyruvate); the assay was made from 3 different sets of experiments and data were represented by mean value ± SE. Different small letters on bar indicate significant at *p* ≤ 0.5.

At a saturated (4.0 mM) malate concentration, the variations in enzyme activity under light and darkness for citrate and succinate were 1.45-, 1.33-, 1.38-, and 1.30-fold increases, as compared to control (Figure 3a). Similarly, oxalate and pyruvate also reduced the activity as inhibitors, irrespective of light and darkness, and they recorded 8 and 22% and 12 and 19% inhibitions as compared to the control, respectively. A compatible trend was almost found when the enzyme activity was recorded using limiting (0.01 mM) malate concentration for both activators and inhibitors. The activators promoted the activities by 1.63 and 1.53-fold for citrate and succinate under light compared to darkness (Figure 3b). Leaf discs incubated in darkness recorded reduced activity when measured in the presence of oxalate and pyruvate as inhibitors, and this was 42 and 43% less under darkness compared to light, respectively.

The behavior of the enzyme in terms of kinetics revealed the possible changes in maximum activity (V_max_) and enzyme–substrate affinity (K_m_), which depicted the impact of salinity (Table 1). Thus, an increase in the K_m_ under salinity by 1.36-fold compared to the control in darkness may suggest the retarded affinity of the enzyme to the substrate. On the contrary, light had slightly overcome the impact of salinity on the enzyme, both by increasing V_max_ and decreasing K_m_. The change in K_m_ under light was 20% decreased compared to darkness, and the increase in V_max_ by 1.36-fold is a clear indication of the effect of salinity.

### 3.3. Effects of Reducing Agent on Activation of Regulatory Properties of NADP-ME under Saturated and Limiting Malate Concentration

**Figure 4:**
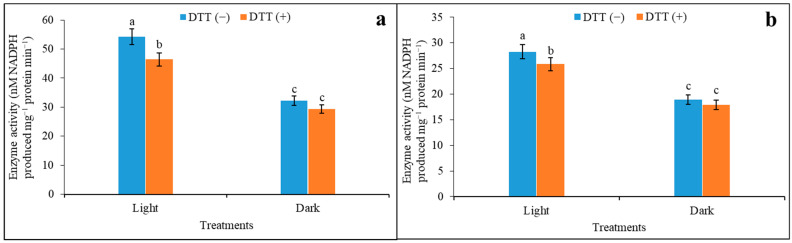
Effect of DTT on NADP-ME activity under light and darkness in 4.0 mM (**a**) and 0.01 mM (**b**) malate concentration. The assay was made from 3 different sets of experiments and data were represented by mean value ± SE. Different small letters on bar indicate significance at *p* ≤ 0.5.

Under both 4.0 mM and 0.01 mM malate concentration, the activity of the enzyme was promoted under light by 1.58 and 1.44-fold as compared to the dark when supplemented with DTT (Figure 4a,b). In another set, a proportionate decrease in activities by 9% under dark when the leaf disc was incubated with DTT under 4.0 mM malate concentration was seen. Leaf discs assayed for enzyme activity under 0.01 mM malate were also inhibited by DTT by 9% in light. The activity was also suppressed under dark compared to light conditions, irrespective of DTT incubation in both malate concentrations. The changes in enzyme activity in the presence or absence of DTT and their impact on light and dark through saturated and limiting malate concentrations were depicted in Table 2, expressed as L/D.

### 3.4. Variability of Polymorphism of NADP-ME under Salinity

**Figure 5:**
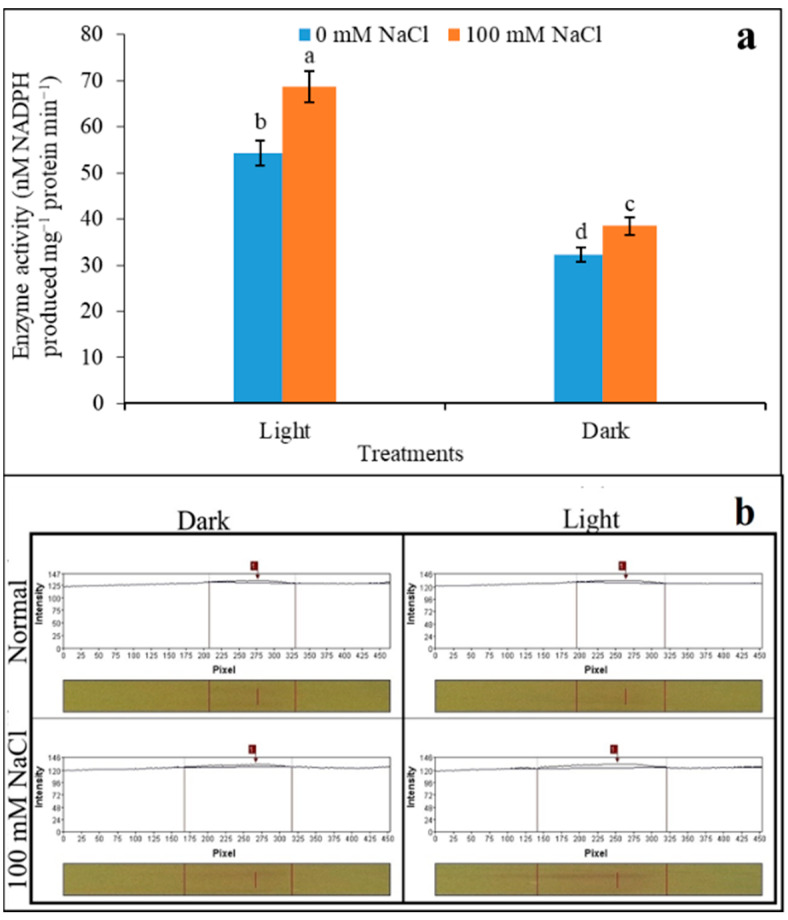
NADP-ME activity in vitro (**a**) and *in-gel* staining (**b**) in saturated condition (4.0 mM malate) under salinity (0 and 100 mM NaCl). The assay was made from 3 different sets of experiments and data were represented by mean value ± SE. Different small letters on bar indicate significance at *p* ≤ 0.5.

The activity of NADP-ME was observed as being upregulated, irrespective of light and darkness, when assayed from salinity. The light-induced activity was over expressed with a salinity induction of 1.26-fold under light (Figure 5a). Contrarily, darkness recorded a similar trend, but in comparison to light only 1.19-fold activity was overexpressed. *In-gel* activity of NADP-ME recorded a significant variation through the treatments. The activity with respect to light- and dark-adapted samples under salinity showed hardly any significant variation in polymorphism (Figure 5b). The band intensities developed with NBT staining showed a single band irrespective of the control and 100 mM NaCl showed a minimum variation in light. Although the number of bands did not vary irrespective of any treatments, light had a dense band in intensities representing protein overexpression. Moreover, leaves had also responded to salinity for a minimum level of overexpression for the enzyme protein. Therefore, with polymorphism, the expression of NADP-ME would be lenient to any salt impact (if any) through light induction.

### 3.5. Chlorophyll Content, Hill Activity, and Sugar Content

**Figure 6:**
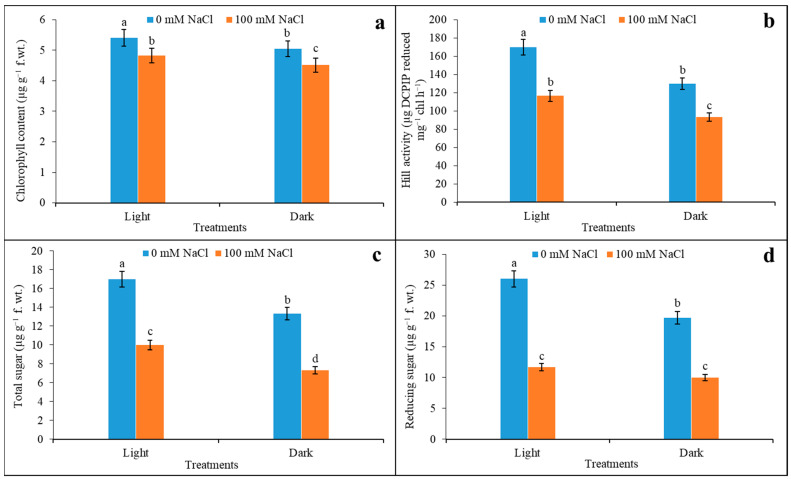
Changes in chlorophyll content (**a**), Hill activity (**b**), total sugar (**c**), and reducing sugar (**d**) content in light and darkness under salinity (0 and 100 mM NaCl). The assay was made from 3 different sets of experiments and data were represented by mean value ± SE. Different small letters on bar indicate significance at *p* ≤ 0.5.

The 100 mM salinity downregulated photosynthetic parameters, such as the chlorophyll content of plants, compared to the control. Under salinity, the maximum decline in total chlorophyll content was reduced by 11% in light as compared to the control (Figure 6a). Although under dark conditions, leaves reduced the chlorophyll content by 11% under salinity, this significantly varied from light under the same condition. The activity of the chlorophyll in terms of the Hill reaction that otherwise denotes the PSII functioning is evident in Figure 6b. The changes in Hill activity were expressed in µg DCPIP reduced by unit of chlorophyll. It demonstrated a distinct variation when light-adapted leaves were compared with darkness. The leaves reduced the activity by 24% under darkness compared to light exposure. Salinity had reduced the activity both for light and darkness by 31 and 28% compared to the control (0 mM NaCl). It is also noted that the impact of the darkness was more pronounced when the leaves were treated with 100 mM NaCl. From the profiles of carbohydrates, both total and reducing sugar declined when faced with salinity. Thus, the variation in total sugar under light and darkness was significant when compared with salinity against the control (Figure 6c,d). The reduced contents of total sugar of 41% in light and 45% in darkness with salinity as compared to the control and almost-compatible reducing sugar were observed, where salinity limited the concentration by 55% under light, and 49% under dark conditions.

### 3.6. Na^+^/K^+^, Proline Content, Malic Acid, and Pyruvic Acid Content under Salinity

**Figure 7:**
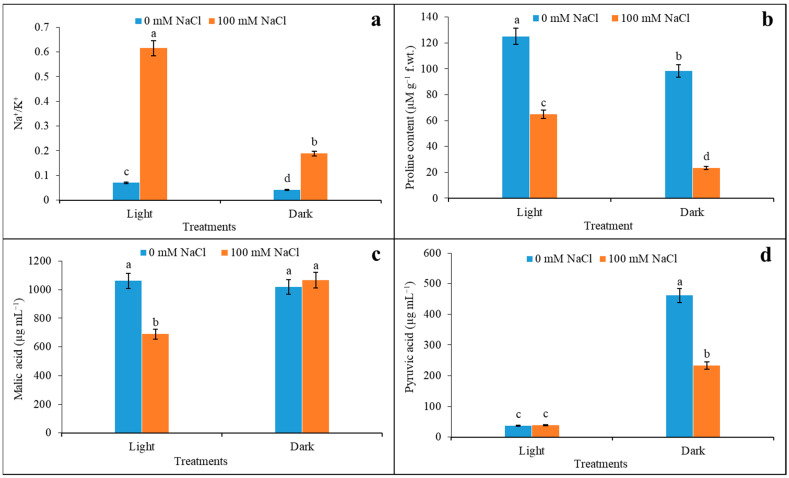
Changes in Na^+^/K^+^ (**a**), proline content (**b**), malic acid (**c**), and pyruvic acid (**d**) content in light and darkness under salinity (0 and 100 mM NaCl). The assay was made from 3 different sets of experiments and data were represented by mean value ± SE. Different small letters on bar indicate significance at *p* ≤ 0.5.

The effect of 100 mM salinity had a direct effect on the tissue concentration of ions where Na^+^ and K^+^ were significantly varied compared to the control (Figure 7a). The membrane permeability of the tissue allowed for the maximum accumulation of Na^+^ by 6.6-fold compared to the control in light, where a depletion of K^+^ by 25% was recorded under the same condition. As expected, the ratio of Na^+^ to K^+^ under salinity had a distinct variation as a function of light and darkness. The indication of salt stress in the plant was demarcated by a significant accumulation of proline concentration; however, this was less than for the control both under light and darkness. Still, the proline was reduced maximally under darkness by 76%; this reduction was 48% under light when assayed from leaves with salinity (Figure 7b). On the other hand, plants accumulated organic acids in a more varied manner when extracted from 100 mM NaCl as a function of light and darkness. It is interesting to note that organic acids related to photosynthesis are more sensitive to 100 mM salinity stress as compared to the control (Figure 7c). Still, malic acid content declined under salinity conditions by 35% compared to the control, however, only under light. Contrarily, in dark conditions, the impact of variation in acid content even under salinity was not significant. On the other hand, the accumulations of pyruvate are significantly varied through light and darkness when exposed to salinity (Figure 7d). Plants recorded a distinct downregulation of pyruvate under 100 mM (50%) as compared to 0 mM NaCl, however, under darkness. Interestingly, under light, there were no changes in acid content recorded, irrespective of NaCl concentration.

## 4. Discussion

The present work hypothesized about the regulations of NADP-ME with its redox status under light and darkness, and how that corroborated with the sensitivity to salinity. In the results, the possible changes in redox that can moderate the NADP-ME activity influencing physiological attributes to salinity tolerance were revealed. In oligomeric enzyme structures, there are possible domains where light-induced redox changes are an absolute requirement for the activation of enzymes in the Calvin cycle [22]. A light-induced sulfohydral (-SH) group is the key factor in the regulation of Calvin cycle enzymes including phosfoenol pyruvate carboxylase (PEPC), glyceraldehyde-3-phospahte dehydrogenase, NADP-ME, etc. [23]. The malate metabolism could replenish the NADPH + H^+^ for Calvin cycle enzymes [24]. Under salt stress and induced stomatal regulation, adequate CO_2_ entry and its downstream assimilation are limited. The NADP-ME activity can overcome the limited substrate (CO_2_ or HCO_3_^−^) to induce a CO_2_ concentration mechanism. The regulation of NADP-ME activities varies with light and darkness, which relates to malate metabolism and ion homeostasis under abiotic stresses [25]. Photosynthetic light flux brings about the conformational changes of the membrane and that is dependent on reduced redox for a proton motive force [26]. There exists a synergistic activity between PEPC and NADPH-MDH that is required for the enrichment of CO_2_ in bundle sheath for the carboxylation of rubisco [27]. Therefore, a stringent regulator of malate oxidative decarboxylation is the control valve for carbon assimilation with NADPH + H^+^. Thus, under salinity and water stress, the NADP-ME induced CO_2_ pump is operative and compensates CO_2_ in cases of impeded carboxylation. The accumulation of CO_2_ for rubisco activation is equally justified with a higher activity of NADP-ME in the present experiment, where it maintains the malic acid to pyruvic acid ratio. Salinity decreased the malate content, which causes induced stomatal closure resulting in a limitation for the Calvin cycle [28]. Under darkness, contrarily, malic acids might have less access to Calvin cycle residues under inadequate light-generated NADPH in photosystem II (PSII). Pyruvic acid in the present experiment was observed less under salinity, but was induced under darkness, and this would be allocated more for stress metabolites rather than Calvin cycle intermediates [29]. The darkness may aggravate salinity effects by inactivating PEPC with de-phosphorylation. Light could overcome salinity with the replenishment of organic acids that activate Calvin cycle enzymes by regulating redox [30].

Osmotic adjustment under water deficit stress also participates in NADP-ME activity. Proline content in durum wheat and NADP-ME activity were correlated for osmoregulation [8]. Proline biosynthesis became the function of light/darkness that also moderated NADPH production, as recorded in the present experiment. Salinity induced osmotic stress, which leads to the accumulation of more proline, glycine betaine, etc., depending on the activity of the proline biosynthetic enzyme, Δ-1-pyrroline-5-carboxylate synthase (ΔP5CS). The ΔP5CS is a light-dependent enzyme that is regulated by ABA and salt stress [31]. Proline biosynthesis with its light dependent induction through ΔP5CS by salt stress is inhibited in dark-adapted plants. The ABA induces ΔP5CS activity more under light, which is overcome by downregulation of the enzyme under dark conditions [31]. Therefore, proline biosynthesis might not be only an indication of salinity stress but also may symbolize the ratio of NADPH to NADP as a redox signaling residue.

Photo-activation for NADP-ME in C_4_ plants is based on light-induced thiol oxidation, which is reported to be the predominant factor [32]. We have evaluated the malic enzyme sensitivity under varying concentrations (saturated and limited) of malic acid. Interestingly, as noted earlier, light illumination may increase the cellular concentration of pyruvic acid in the bundle sheath, with an optimum value in maize [33]. This is accompanied by enzyme activities releasing CO_2_ levels over ten-fold compared to the ambient concentration. Therefore, HCO_3_^−^ concentration becomes optimum for PEPC and leads to more accumulation of oxaloacetate following malate. This is the reason for adoption, function, and modulation of NADP-ME under elevated levels of CO_2_ and pyruvate [34]. In the present experiment, the activity of NADP-ME was observed with a direct relationship in response to HCO_3_^−^ in a leaf disc. The increased HCO_3_^−^ concentration (decreased pH) in exchange for the hydroxyl ion (OH^−^) would be another factor for PEPC in the conversion of oxaloacetic acid (OAA). Under stimulated photosynthetic irradiance, the activities of PEPC, NADP-MDH, NADP-ME, and phosphoenolpyruvate carboxy kinase (PEPCK) are induced to develop a steady-state malate concentration in the bundle sheath. Thus, in the experiment, the leaf discs of maize maintained a firm rise in NADP-ME activity within 1200 µE m^−2^ s^−1^ light intensities against dark conditions for both saturated (4.0 mM) and limiting (0.01 mM) malate concentrations. Salinity undoubtedly increases the higher bioaccumulation of HCO_3_^−^ along with sodium ions (Na^+^) from soil in exchange for potassium (K^+^). The ion homeostasis for K^+^/Na^+^ is key factor in salt toxicity, which impedes the water relationship, photosynthetic gas exchange, solute translocation, etc. The loss of K^+^ causing stomatal closure led to a decrease in pH, favoring NADP-ME for optimum activity [35]. This is more prevalent when plants experience higher illumination causing stomatal pH to be more alkaline. This is validated herein, where 100 mM NaCl induced the activity of NADP-ME to be several folds higher; this may indicate light sensitization, however, under a saturated malate concentration. Therefore, the activity of NADP-ME would be complimented by light-induced redox changes, -SH/-S-S-, as well as under alkalinity [36]. Polymorphic gene expression for NADP-ME, as deduced through *in-gel* activity staining, also confirmed the light-induced NaCl sensitivity in the present experiment. The variation in protein contents and its function in decarboxylation activity subjected to salinity may justify the importance of NADP-ME in contributing to stress tolerance on a photosynthetic level [37].

For the characterization of the enzyme, the activity of NADP-ME on the basis of protein sensitivity to activators and inhibitors was assessed in the present study. Under 1200 µE m^−2^ s^−1^ of light flux, a significant downregulation is caused, which is inhibited by pyruvate with an increase in K_i_ for pyruvate [38]. The inhibition of the conversion of NADP-ME to pyruvate/OAA is almost similar to other photosynthetic enzymes in C_3_ and C_4_ plants. Contrarily, the enzyme activities become induced with citrate/succinate, which possibly compensates for the inadequate redox or kinetic property changes experienced under light and darkness. The activation and deactivation of NADP-ME are interesting with reference to both light/darkness and pH sensitivity. A fall in sensitivity is due to feedback inhibitions on illumination, as found for PEPC, nitrate/nitrite reductase, and sucrose phosphate synthase with dephosphorylation by light induction [39]. Other light regulatory enzymes are based on changes in the thiol group(s) with reduction by NADPH/reduced ferredoxin (Fd_red_). In the present experiment, protein decreased the sensitivity to feedback inhibition with dithiothreitol (DTT) under light and darkness. Dark-adapted leaf discs are more sensitive to inhibitors; however, they are equally responsive to DTT to reduce functional groups on the active site of the enzyme [40]. Therefore, the post translational modification could be justified by reversible changes in redox as a function of light and darkness. This is applicable for the oligomeric enzymes by the acceptance of electrons from reducing equivalents (NADPH + H^+^ and Fd_red_) which causes activation and deactivation.

There are few possibilities of post-translational changes for modulations of enzyme activity where phosphorylation–dephosphorylation is predominant [41]. Salinity-induced redox changes in plant tissues are also responsible for enzyme kinetic properties such as maximum activity (V_max_), Michaelis–Menten constant (K_m_), etc., with which changes in activities can be ascertained. In the present experiment, the observed changes for K_m_, possibly through the modification of functional groups, indicated the impact of salinity on NADP-ME activity vis-à-vis photosynthetic oxidative decarboxylation. Under salinity, the increased value of K_m_ undoubtedly suggested the lesser affinity of malate as a substrate to the enzyme, as indicated in citations [42]. Earlier studies with C_4_ plants confirm that a decrease in K_m_ but an increase in V_max_ are the outcomes of enabled formation and the breakdown of the enzyme substrate complex, manifesting the upregulation of reaction rate which is hindered under salinity [43]. Besides the phosphorylation–dephosphorylation of the enzyme protein, there are other fates of enzyme conformation where the reduction–oxidation of dithiols (-S-S-) is important. As already reported, NADP-ME is extremely light sensitive so the reduction of dithiols when incubated with reducing agents such as DTT (as exercised in present experiment also) could diminish the light-induced enzyme activity, even in the dark also. Seven cysteinyl residues are seated on oligomeric units of NADP-ME, as identified in maize, and are under the control of the Fd/Trx system [44]. Therefore, the activity of NADP-ME and its regulation are more complex, with changes in enzyme kinetics through redox homeostasis being influenced under light.

The Hill activity is a direct indication of PSII functioning which is sensitive to both light reaction as well as the metabolism of the Calvin cycle. Undoubtedly, the attenuation of PSII by insufficient irradiation would be a direct limiting factor for the energy transfer mechanism between two photosystems [45]. This exactly was satisfied in the present experiment where darkness-adapted leaves had reduced activity accompanied by low chlorophyll content. Meanwhile, salinity is more vulnerable for the photosystem rather than irradiance since the impact of sodium causes destabilization of ionic concentration in the stroma [46]. This affects the H^+^ gradient over the chloroplast membrane between the thylakoid and lumen, causing inadequate functioning of photosynthetic phosphorylation. In addition, most of the Calvin cycle enzymes are moderately sensitive to the optimum concentration of Mg^2+^, which is depleted with acquisition of Na^+^ in salinity stress [47]. Therefore, salinity compounded with darkness would be the key to the inadequate activity of Calvin cycle enzymes where NADP-ME would be predominant, as was also found in the present experiment.

The consumption of carbohydrates by plants is mostly for two processes: first, to support the reduced carbon for bio-residue generation, and second, to replenish the compatible solutes [48]. The last one is more proportionate to the adjustment of the osmotic relationship under salinity or related stress, as is also observed in the present experiment. Now, the question is regarding carbohydrate utilization and its support for plant tolerance when recorded during light induction. It is interesting to note that light has a significant role in the interactions of salinity stress because of its inducing power to develop oxidative redox [49]. In plants, adequate citations could strengthen the role of higher irradiance to develop ROS under NaCl and xenobiotic stress and thereby to induce antioxidation capacity with the development of non-enzymatic antioxidants from carbohydrate flux. The light-induced overproduction of compatible solutes and ROS-quenching residues is also encouraged for salinity tolerance in maize and rice [50]. Therefore, all sugar fractions would be indicative of probable stress tolerance with the support of light exposure compared to dark. It is also evident from the proportion of sugars from the photosynthetic flux, which is also dependent on the reactions of decarboxylation in malate metabolism [51].

## 5. Conclusions

The NADP-ME activity in plants is well established for its regulation under photosynthetically active radiation through light/dark-induced activation/deactivation. However, under saline soil, when roots absorbed Na^+^ and it was translocated into mesophyll cells, photosynthetic activities were disturbed. Still, how salinity could impact the CO_2_ concentration mechanism with the induction of NADP-ME is less documented with regards to cereal crops. In this aspect, the present findings may cultivate new insights compared to the existing literature on the same topic, however with reference to maize plants. Conclusively, it is evident that a minimum increase in NADP-ME activity from maize leaves, as a function of light induction, is based on changes in regulatory properties. This is also coordinated with the sensitivity of the enzyme to activators and inhibitors. Importantly, the redox changes in projected thiol groups under the reducing equivalent of NADPH + H^+^ or Fd_red_ would be possible candidates, as evidenced from the application of the reducing agent (DTT) in this experiment. Moreover, salinity-induced upregulation of enzyme activity is also another line of evidence where C_4_ plants expressed sustainability by their retrieval of photosynthetic oxidative decarboxylation. The released CO_2_ in this reaction is quite enough to replenish the reduced carbon in many biosynthetic pathways such as osmolytes, etc. Therefore, the major metabolite leading to the function of the tissue-specific CO_2_ concentrating mechanism is malate in C_4_ plants. Utilizing NADP-ME NADP-MDH plants can demonstrate the utilization of malate as a source of carbon residue in adaptation to environmental changes. Therefore, an introduction or over-expression of C_4_-specific NADP-ME pathways in the plant system would be effective for malate metabolism and thereby may establish tolerance potential in plants. More studies at the molecular level are invited in order to investigate the expression potential of NADP-ME with other Calvin cycle enzymes in photosynthetic pathways to promote overall plant sustainability under stressful conditions.

## Figures and Tables

**Table 1:** Kinetic properties through enzyme–substrate affinity (K_m_) and V_max_ of NADP-ME from leaf discs when illuminated with 1000–1200 µE m^−2^ s^−1^ under 2 mM bicarbonate. The assay was made from 3 different sets of experiments and data were represented by mean value ± SE. K_m_ = Michaelis constant, V_max_ = maximum rate of reaction.

Treatments	K_m_ (mM/L)		V_max_ (nM min^−1^ mg^−1^ Protein)	
	Light	Dark	Light	Dark
0 mM NaCl	0.09 ± 0.006 ^a^	0.11 ± 0.006 ^ab^	61.89 ± 1.33 ^a^	43.89 ± 1.80 ^b^
100 mM NaCl	0.12 ± 0.01 ^b^	0.15 ± 0.01 ^c^	64.46 ± 2.10 ^a^	47.1 ± 1.70 ^bc^

Different small letters indicate significant at *p* ≤ 0.5.

**Table 2:** The effects of 10 mM DTT in incubation mixtures under light- (1000–1200 µE m^−2^ s^−1^) and darkness-adapted 2 mM HCO_3_^−^ pre-incubated leaf discs for 35 min through saturated and limiting malate concentration. The activity ratio under light and (L/D) was calculated from 3 different sets of experiments and data were represented by mean value ± SE.

Treatments	Saturated (4.0 mM) Malate Concentration			Limiting (0.01 mM) Malate Concentration		
	Light	Dark	L/D	Light	Dark	L/D
DTT (−)	54.32 ± 1.12 a	32.24 ± 1.36 c	1.68	28.28 ± 0.64 a	18.92 ± 0.90 c	1.49
DTT (+)	46.42 ± 1.52 b	29.32 ± 0.77 c	1.58	25.83 ± 0.78 b	17.89 ± 1.01 c	1.44

Different small letters indicate significant at *p* ≤ 0.5.

## Data Availability

All data are available in this paper.
